# Aktuelle Entwicklungen in der Behandlung neuroendokriner Tumoren

**DOI:** 10.1007/s00117-024-01303-2

**Published:** 2024-04-22

**Authors:** Barbara Kiesewetter-Wiederkehr, Philipp Melhorn, Christian Scheuba, Markus Raderer

**Affiliations:** 1 Klinische Abteilung für Onkologie, Universitätsklinik für Innere Medizin I, Medizinische Universität Wien, Waehringer Guertel 18–20, 1090 Wien, Österreich; 2 Universitätsklinik für Allgemeinchirurgie, Medizinische Universität Wien, Wien, Österreich

**Keywords:** Neuroendokrine Tumoren, Somatostatin-Analogon, Peptidrezeptor-Radionuklid-Therapie, Everolimus, Chirurgie, Neuroendocrine tumors, Somatostatin analogs, Peptide receptor radionuclide therapy, Everolimus, Surgery

## Abstract

**Hintergrund:**

Gut differenzierte neuroendokrine Tumoren (NET) sind seltene Malignome, die klinisch sehr heterogen sind. Entsprechend ist auch ihre Behandlung komplex und von diversen Faktoren abhängig. Mit den heute verfügbaren Therapien ist die Prognose oft günstig.

**Ziel der Arbeit:**

Dieser Artikel soll einen Überblick über die aktuellen Therapiestrategien bei NET geben und dabei auf die wichtigsten NET-Lokalisationen eingehen.

**Methoden:**

Zu diesem Zweck wurden die aktuellen europäischen Leitlinien und die einschlägige Literatur zur Behandlung von NET zusammengefasst.

**Ergebnisse:**

Das Therapiespektrum ist ausgesprochen breit: Bei den NET des Magens/Duodenums, der Appendix und des Rektums ist oft die endoskopische respektive chirurgische Entfernung ausreichend, und metastasierte Tumoren sind selten. NET des Pankreas, des Dünndarms und der Lunge sollen in frühen Stadien auch einer potenziell kurativen Resektion zugeführt werden. Im metastasierten Stadium haben lokale Therapiemaßnahmen wie Chirurgie und Leber-Tumorembolisation ebenfalls einen Stellenwert. Zuletzt gab es viele Fortschritte hinsichtlich der medikamentösen Therapie, wobei insbesondere Somatostatin-Analoga (Octreotid und Lanreotid), ein mTOR-Inhibitor (Everolimus) und ein Tyrosinkinase-Inhibitor (Sunitinib) eingesetzt werden. Ferner stellt die Peptidrezeptor-Radionuklid-Therapie (PRRT) ein wichtiges Verfahren dar. Auch die klassische Chemotherapie ist in einigen Fällen indiziert.

**Diskussion:**

Inzwischen gibt es viele effektive Therapien für NET. Es ist wichtig, im interdisziplinären Management für jeden Patienten und jede Patientin die richtige Therapie zum richtigen Zeitpunkt auszuwählen.

## Abstract (en)

**Background:**

Well-differentiated neuroendocrine tumors (NET) are rare malignancies that are clinically very heterogeneous. Accordingly, their treatment is also complex and dependent on various factors. With currently available systemic therapies, the prognosis is often favorable.

**Objectives:**

This article aims to provide an overview of current treatment strategies for NET, addressing the most important NET locations.

**Methods:**

The current European guidelines and further relevant literature on the treatment of NET were reviewed for this purpose.

**Results:**

The therapeutic spectrum for NET is extremely broad: For NET of the stomach/duodenum, appendix, and rectum, endoscopic or surgical resection is often sufficient, and metastatic tumors are rare. NET of the pancreas, small intestine and lung should also undergo potentially curative resection in the early stages. In the metastatic stage, locoregional treatments such as surgery and liver tumor embolization play a role. Major advances have been made in drug therapy, with somatostatin analogs (octreotide and lanreotide), an mTOR inhibitor (everolimus), and a tyrosine kinase inhibitor (sunitinib) being widely used. Peptide receptor radionuclide therapy (PRRT) is also an invaluable option. In some cases, classic chemotherapy is indicated.

**Conclusions:**

Many effective therapies are now available for NET. It is important to select the right therapy at the right time for each patient through interdisciplinary management.

In den vergangenen Jahren gab es einige Entwicklungen hinsichtlich der Behandlung von neuroendokrinen Tumoren (NET). NET sind eine besonders heterogene Gruppe von Malignomen, deren Management oft komplex und meist nur interdisziplinär und multimodal möglich ist. Diese Diversität zeigt sich auch darin, dass die European Neuroendocrine Tumor Society (ENETS) im Jahr 2023 sechs neue „ENETS guidance papers“ publiziert hat. Dieser Übersichtsartikel soll daher alle praxisrelevanten Erkenntnisse zur Behandlung der NET anhand der aktuellen Leitlinien darstellen.

Im Folgenden werden dabei nur die gut differenzierten NET behandelt, die sich pathologisch, therapeutisch und prognostisch grundlegend von den schlecht differenzierten neuroendokrinen Karzinomen (NEC) unterscheiden [19]. Für die verschiedenen anatomischen Ursprungsorte von NET gibt es teils eigene histologische Klassifikationen, wobei sich bei den gastrointestinalen und pankreatischen NET eine Einteilung in 3 Tumorgrade (NET G1-G3) durchgesetzt hat [19]. Diese Unterteilung basiert auf dem Ki67-Index bzw. der Mitoseanzahl/mm^2^ (Ki67-Index bei NET G1 < 3 %, bei NET G2 3–20 % und bei NET G3 > 20 %) und ist prognostisch bedeutsam [19].

## NET des Pankreas

Neuroendokrine Tumoren des Pankreas (panNET) sind in ihrem klinischen Bild äußert variabel und das initiale Management hängt von mehreren klinischen Faktoren ab. Dies sind zum einen die Lokalisation und Anzahl der Tumoren, Fernmetastasierung und Tumorlast sowie der Tumorgrad und die Expression des Somatostatin-Rezeptors (vor allem SSTR2) [9]. Zum anderen können panNET in ca. 10–30 % mit diversen funktionellen Syndromen (Hypoglykämie bei Insulinom oder Zollinger-Ellison-Syndrom bei Gastrinom) assoziiert sein bzw. im Kontext von hereditären Syndromen wie MEN1 (multiple endokrine Neoplasie Typ 1) oder beispielsweise VHL-Syndrom (Von-Hippel-Lindau-Syndrom) auftreten [17].

Bei lokalisierten panNET nimmt die Chirurgie eine wichtige Rolle ein, wobei es einige Besonderheiten gibt, je nachdem, ob ein hormonelles Syndrom (F-panNET) vorliegt oder nicht (nichtfunktioneller Tumor, NF-panNET; [8, 11]). Für NF-panNET ≥ 2 cm gibt es eine starke Empfehlung für eine kurativ intendierte Resektion (bestenfalls minimal-invasiv), wobei meist eine Standard-Pankreatektomie (Duodenopankreatektomie oder distale Pankreatektomie) mitsamt Lymphadenektomie empfohlen wird [11, 17]. Lokal fortgeschrittene Tumoren (T3 und T4; > 4 cm oder Hinauswachsen über das Pankreas) können auch resektabel sein [11]. Bei lokalisierten asymptomatischen NF-panNET ≤ 1 cm (bzw. ≤ 2 cm unter gewissen Umständen) ohne Dilatation des Ductus pancreaticus sollte eine aktive Überwachung stattfinden [11, 17]. Im Gegensatz dazu sollen bei F‑panNET auch kleine (≤ 2 cm) lokalisierte Tumore chirurgisch entfernt werden [17]. Ferner haben lokalisierte Insulinome häufig ein eher benignes Verhalten und seltener Lymphknotenmetastasen, sodass grundsätzlich parenchymsparende Resektionen (ohne formale Lymphadenektomie) empfohlen werden [8]. Gastrinome hingegen zeigen häufig Lymphknotenmetastasen, können in Pankreas und im Duodenum auftreten und zu etwa einem Viertel im Kontext von MEN1, weswegen hier das chirurgische Vorgehen im lokalisierten Stadium etwas anders ist (systematische Lymphadenektomie bei Resektion des Primums; [8]).

Im metastasierten Setting ist die SSTR-Expression therapieentscheidend; Tab. 1. SSTR-positive Tumoren, besonders wenn sie langsam wachsen (Ki67-Index < 10 %), können initial mit monatlichen Depotinjektionen der langwirksamen Analoga des Hormons Somatostatin häufig ausreichend gut behandelt werden – also mit einem der beiden Somatostatin-Analoga (SSA) Lanreotid Autogel oder Octreotid LAR. Der tumorstabilisierende Effekt von Lanreotid konnte in der Phase-III-Studie CLARINET gezeigt werden, in der es zu einer signifikanten Verlängerung des progressionsfreien Überlebens (PFS) verglichen mit Placebo kam [5]. Für Octreotid gibt es randomisierte Daten für Dünndarm-NET (Midgut-NET) aus der PROMID-Studie, wobei man allgemein von einem Wirkstoffklassen-Effekt ausgeht und beide SSA als gleichwertig angesehen werden [16, 17, 21]. Insgesamt lässt sich feststellen, dass SSA ein äußerst günstiges Nebenwirkungsprofil haben (am häufigsten sind gastrointestinale Beschwerden) und diese Medikamente auch bei langjähriger Dauertherapie meist gut vertragen werden [5].

**Tab. 1:** Übersicht über die aktuellen Therapieoptionen bei metastasierten NET gemäß ENETS und ESMO (siehe auch [2, 8, 10, 11, 15–17, 20])

NET-Lokalisation	Tumorgrad	Charakteristika	Erstlinie	Zweitlinie	Drittlinie
*Pankreas*	NET G1/2	Langsames Wachstum SSTR-positiv Ki67 <10 %	SSA Lokale Therapien^1^	STZ/5-FU CAPTEM Everolimus Sunitinib	PRRT
	NET G2	Ki67 >10 %	STZ/5-FU CAPTEM Everolimus Sunitinib	PRRT	–
	NET G3	–	CAPTEM STZ/5-FU	Everolimus Sunitinib	PRRT
	–	SSTR-negativ	STZ/5-FU CAPTEM Everolimus Sunitinib	–	–
*Dünndarm*	NET G1/2	SSTR-positiv Ki67 <10 %	SSA Watch-and-wait Lokale Therapien^1^	PRRT	Everolimus
	NET G2	Ki67 >10–15 %	Everolimus	PRRT	FOLFOX CAPTEM
	–	SSTR-negativ	Lokale Therapien^1^	Everolimus	Interferon-alpha
*Lunge*	Typische Karzinoide	Oder langsam wachsende Karzinoide	Watch-and-wait SSA Lokale Therapien^1^	–	–
	Atypische Karzinoide	Oder eindeutig progrediente Karzinoide	Everolimus Temozolomid-basiert	Platinum-basiert PRRT Interferon-alpha	–
*Kolon/Rektum*	NET G1/2	SSTR-positiv wenig Tumorlast Ki67 <10 %	SSA	Everolimus PRRT Lokale Therapien^1^	TKI
	NET G1/2	SSTR-positiv hohe Tumorlast Ki67 >10 %	Everolimus Chemotherapie PRRT	TKI Lokale Therapien^1^	–
	NET G1/2	SSTR-negativ	Everolimus	Lokale Therapien^1^ Chemotherapie	–
	NET G3	–	CAPTEM	Wenig Tumorlast: Everolimus, TKI, PRRT Hohe Tumorlast: Oxaliplatin-basierte Chemotherapie	–
*Magen/Duodenum*	Es liegen kaum Studienergebnisse vor, mögliche Optionen sind SSA, PRRT, Everolimus und für NET G3 Chemotherapie				
*Appendix*	Aufgrund der Seltenheit gibt es kaum Daten zur systemischen Therapie, bei Metastasierung in der Regel an der Therapie enteraler NET orientiert				

*NET* neuroendokriner Tumor, *SSTR* Somatostatin-Rezeptor, *SSA* Somatostatin-Analoga, *STZ/5-FU* Streptozotocin/5-Fluoruracil, *CAPTEM* Capecitabin/Temozolomid, *PRRT* Peptidrezeptor-Radionuklid-Therapie, *FOLFOX* 5-Fluoruracil/Leucovorin/Oxaliplatin, *TKI* Tyrosinkinase-Inhibitor

^1^Lokale Therapien sind bei leberbetonter Metastasierung eine Option

Die Peptidrezeptor-Radionuklid-Therapie (PRRT) ist eine rezeptorbasierte nuklearmedizinische Therapieoption, die häufig bei Dünndarm-NET zum Einsatz kommt (meist 4 Zyklen alle 8 Wochen) [25]. Dabei wird beispielsweise das Radionuklid ^177^Lutetium mittels Chelator DOTA an ein Somatostatin-Analogon gelinkt, sodass es zielgerichtet an Somatostatin-Rezeptoren binden kann [25]. Für SSTR-positive panNET gibt es limitierte prospektive Phase-II-Evidenz von der OCLURANDOM-Studie (PRRT oder Sunitinib) und der retrospektiven Studie NETTER‑R, weswegen diese Therapie in der Zweitlinie für Patient/-innen mit panNET G1/G2 als eine Option empfohlen wird [11]. Bei gastroenteropankreatischen (GEP) NET G2/G3 wird derzeit die PRRT als Erstlinientherapie untersucht (NETTER‑2, NCT03972488) bzw. mit Chemotherapie/Everolimus verglichen (COMPOSE, NCT04919226).

Eine weitere systemische Therapieoption (bei SSTR-positiven panNET nach SSA bzw. bei SSTR-negativen auch upfront) ist der Tyrosinkinase-Inhibitor (TKI) Sunitinib, der ebenfalls in einer Phase-III-Studie (Raymond et al.) zu einer PFS-Verlängerung (vs. Placebo) geführt hat (Hazard-Ratio/HR 0,42; 95 % Konfidenzintervall [KI] 0,26–0,66) und ferner zu einer objektiven Ansprechrate von ca. 10 % [18]. Breiter eingesetzt (auch bei anderen NET-Lokalisationen) wird der mTOR-Inhibitor (mammalian Target of Rapamycin-Inhibitor) Everolimus, der in der Phase-III-Studie RADIANT‑3 bei progressiven panNET G1/G2 in einer Verbesserung des PFS resultiert hat (medianes PFS von 11,0 Monaten vs. 4,6 Monaten mit Placebo) [30]. Weil es de facto keine robusten Vergleichsdaten der einzelnen Therapien gibt, wird die Wahl der Zweitlinientherapie (Tab. 1) meist individuell auf die Patient/-innen abgestimmt.

Bei „bulky disease“ respektive symptomatischen oder schnell wachsenden NF-panNET wird von den ENETS-Leitlinien eine Chemotherapie (in der Erstlinie) bevorzugt, wobei viele Faktoren die Therapieentscheidungen beeinflussen können (Patient/-innen-Charakteristika, Therapieziel, Metastasierungsausmaß, Lokaltherapien etc.) [11]. Seit über 40 Jahren wird das Alkylans Streptozotocin (STZ) bei panNET angewendet, oft in Kombination mit dem Antimetaboliten 5‑Fluoruracil (5-FU; Capecitabin ist die orale Prodrug davon) [4]. Rezent (2023) wurden die Ergebnisse der ECOG-ACRIN E2211-Studie veröffentlicht, die bei 144 Patient/-innen mit panNET G1/G2 Temozolomid mono mit Capecitabin/Temozolomid (CAPTEM) verglichen hat – mit signifikant besserem PFS sowie numerisch besserer Ansprechrate und Gesamtüberleben bei CAPTEM [12]. Für panNET G3 wird von ENETS derzeit CAPTEM favorisiert [11]. Die ersten Daten der SEQTOR-Studie (Sequenz STZ/5-FU zu Everolimus vs. die umgekehrte Reihenfolge) zeigen ähnliche Ergebnisse für den primären Endpunkt (1-Jahres-PFS1-Rate), aber auch eine höhere Ansprechrate für die Chemotherapie [23]. Die BETTER2-Studie (NCT03351296; Randomisierung zu CAPTEM oder STZ/5-FU und dann zu plus/minus Bevacizumab) könnte zukünftig zur Therapie von panNET aussagekräftige Resultate bringen [4].

Zur symptomatischen Therapie fortgeschrittener F‑panNET mit leberbetonter Tumorlast sollen eine zytoreduktive Operation, ablative (Radiofrequenzablation) oder embolisierende (transarterielle Chemo‑/Radio‑/Embolisation) Verfahren erwogen werden [8]. Die angeführten systemischen Therapien zur Tumorkontrolle/-reduktion führen oftmals auch zu einer guten Symptomkontrolle (allen voran die auf viele gastrointestinale Hormone inhibitorisch wirkenden SSA) [8]. Für spezifische hormonelle Syndrome seien ferner individuelle Vorgehensweisen erwähnt (Insulinom: Diazoxid, Gastrinom: Protonenpumpeninhibitoren, ACTHom: Steroidgenese-Enzyminhibitoren wie Metyrapon oder Ketoconazol) [8]. Everolimus verursacht bei einem Teil der Patient/-innen Hyperglykämien, daher ist bei einem Glukagonom Vorsicht geboten [30]. Daten zu Kombinationstherapien, Immuntherapie (Immuncheckpoint-Inhibitor) und Antikörper-Wirkstoff-Konjugaten (ADC) sind limitiert [11].

## NET der Appendix

Aus onkologischer Sicht unterscheiden sich die Appendix-NET (aNET) stark von den anderen NET-Lokalisationen hinsichtlich ihres klinischen Verlaufs, der oft sehr günstig ist: Meist werden sie im Rahmen einer Appendektomie inzidentell entdeckt (sie stellen > 50 % aller Appendix-Neoplasien dar) und sind damit oft kurativ entfernt [27]. Soweit bezifferbar, zeigen nur ca. 1 % eine Fernmetastasierung, und aNET < 1 cm haben nur in 10–20 % Lymphknotenmetastasen, wobei nicht wirklich klar ist, ob diese Faktoren überhaupt das Gesamtüberleben beeinflussen [27]. Das spiegelt sich auch in den Behandlungsempfehlungen in den aktualisierten ENETS-Leitlinien über aNET wider, die primär chirurgische Fragestellungen und die Nachsorge diskutieren [10].

Der gängige Stratifizierungsfaktor ist die Tumorgröße. Generell wird für Tumoren < 1 cm eine einfache Appendektomie empfohlen, während aNET > 2 cm einer Komplettierungsoperation im Sinne einer rechtsseitigen Hemikolektomie zugeführt werden sollten [10]. Wichtige Daten für die Empfehlungen bezüglich der dazwischenliegenden Gruppe stammen von einer rezenten (2023) retrospektiven europaweiten Analyse, die anhand von 282 Patient/-innen mit komplett resezierten aNET 1–2 cm das Outcome nach Appendektomie alleine vs. Appendektomie mit rechtsseitiger Hemikolektomie oder Ileozökalresektion verglichen hat [14]. Nach einem im Median 13 Jahre langen Follow-up zeigte sich ein ähnliches Gesamtüberleben der beiden Gruppen und kein höheres Rezidivrisiko bei nodal positiven aNET, weswegen diese Studie eine rechtsseitige Hemikolektomie nach kompletter Tumorentfernung allgemein für nicht indiziert beschreibt [14]. Laut ENETS-Leitlinien rechtfertigen gewisse Risikofaktoren wie ein hoher Tumorgrad (neben einer inkompletten Entfernung) eine Komplettierungsresektion [10]. Bildgebende Nachuntersuchungen werden im Anschluss an eine Hemikolektomie nur bei einer Hochrisikokonstellation (> 2 cm, hoher Tumorgrad, Lymphknoten positiv) empfohlen [10]. Es gibt keine Indikation für eine adjuvante Systemtherapie nach der kompletten Resektion von gut differenzierten NET [10]. Die systemischen Therapiestrategien für die ausgesprochen selten metastasierten aNET orientieren sich an den Behandlungsprinzipien der anderen gastrointestinalen NET [10].

## NET gastroduodenalen Ursprungs

Meist spielt auch bei den gastrischen NET (gNET) die systemische Antitumor-Therapie eine untergeordnete Rolle. Die revidierten ENETS-Leitlinien empfehlen für Typ-I-gNET (bei chronisch-atropher Gastritis) mit einer Größe < 1 cm eine endoskopische Überwachung (Ösophagogastroduodenoskopie, ÖGD), bei 1–2 cm eine Form der endoskopischen Resektion und bei > 2 cm eine chirurgische Entfernung [15]. Vor der endoskopischen Resektion sollte meist ein endoskopischer Ultraschall zur Beurteilung der lokalen Invasionstiefe und möglicher Lymphknotenmetastasen erwogen werden, da ein Vorwachsen in die Muscularis propria, suspizierte Lymphknotenmetasten oder andere Risikofaktoren ein radiologisches Staging und meist ein chirurgisches Vorgehen nach sich ziehen sollten [15]. Bei metastasierten oder nichtresezierbaren Typ-I-gNET empfehlen die ENETS-Leitlinien eine SSA-Therapie [15]. Die seltenen Typ-II-gNET (verursacht durch ein Gastrinom) sind im Kontext der meist zugrundeliegenden MEN1 zu behandeln [15]. Die aggressiveren und prognostisch schlechten Typ-III-gNET sind grundsätzlich eine Domäne der Chirurgie (Wedgeresektion bis Gastrektomie) [15]. Die neuen Leitlinien weisen aber auch darauf hin, dass nach einem sorgfältigen Staging und bei günstigen Faktoren (< 1 cm, G1, lokalisiert) eine endoskopische Resektion erwogen werden kann [15].

Bei den duodenalen NET (dNET) ist ebenfalls die Tumorgröße ein therapieentscheidender Faktor. Sehr kleine dNET (< 5 mm) werden in der Regel mittels endoskopischer Resektion entfernt, was teils auch bei einer Größe von 5–10 mm noch möglich ist [15]. Bei höherem Risiko (> 10 mm, über die Submukosa hinauswachsend, G2/G3, funktionelle Symptome) wird generell die chirurgische Entfernung (lokale Resektion bzw. Duodenopankreatektomie) empfohlen [15]. Zum therapeutischen Management metastasierter dNET gibt es kaum spezifische Daten, und die Optionen orientieren sich an denen der anderen NET-Lokalisationen [15].

## NET kolorektalen Ursprungs

Die aktualisierten ENETS-Guidelines zu den kolorektalen NET (crNET) streichen zu Beginn die Ähnlichkeiten (prognostisch) und Unterschiede (pathologisch) zwischen den rechtsseitigen Kolon-NET und den Dünndarm-NET hervor, die beide ihrem embryologischen Ursprung nach unter „Midgut-NET“ zusammengefasst werden [20]. Als solche wurden diese Tumoren in den klinischen Studien PROMID [21], NETTTER‑1 [25], CLARINET [5] und RADIANT‑2 [6] miteingeschlossen und ausgewertet, sodass die Leitlinien auch eine Behandlung entsprechend der Dünndarm-NET empfehlen [20]. Einzelne Patient/-innen mit „Hindgut NET“ – sprich distales Kolon und rektale NET – waren auch in der CLARINET-Studie inkludiert, wobei anhand der geringen Anzahl kein signifikanter Effekt des Somatostatin-Analogons Lanreotid vs. Placebo gezeigt werden konnte [5]. Von der ENETS gibt es eine Empfehlung für SSA in der Erstlinie bei metastasierten crNET G1/G2 mit SSTR-Expression (entsprechend schwächere Empfehlung bei rNET; Tab. 1) [20]. Bei crNET wird bei Progression oder mangelnder SSTR-Expression Everolimus empfohlen [20], weil sich in den Subgruppenanalysen (10–20 % der Gesamtkohorte) von RADIANT‑2 (funktionelle Tumoren) und RADIANT‑4 (nichtfunktionelle Tumoren) ein signifikanter Vorteil für Everolimus gezeigt hat [6, 24]. Es gibt nur wenig spezifische Daten über Chemotherapie (am ehesten CAPTEM oder FOLFOX [5-Fluoruracil/Leucovorin/Oxaliplatin] für schnell fortschreitende NET) und PRRT bei crNET [20].

Zu den frühen Stadien sei noch angeführt: Ob bei der Entfernung von lokalisierten rektalen NET endoskopisch oder chirurgisch vorgegangen werden soll, richtet sich nach dem TN(M)-Stadium bzw. bei Zweiteingriff vor allem nach dem R‑Status und dem Tumorgrad [20]. Kolon-NET sollten üblicherweise chirurgisch angegangen werden, außer bei selektierten Fällen mit einem kleinen G1-NET [20].

## NET des Dünndarms

Neben den NET des Rektums und der Lunge sind die Dünndarm-NET („small intestinal“, siNET) die häufigsten neuroendokrinen Tumoren, wobei sie bei Diagnose in ca. 30 % fernmetastasiert sind [29]. Häufig (> 20 %, je nach Stadium) weisen siNET ein Karzinoidsyndrom auf, sprich Symptome wie Flush (Erröten), Diarrhoe und Bronchokonstriktion bzw. langfristig das Karzinoid-Herz-Syndrom [7, 17]. Eine umfassende ENETS-Leitlinie zum Thema Karzinoidsyndrom wurde 2022 veröffentlicht [7]. Für siNET allgemein gibt es zum Verfassungszeitpunkt des Manuskripts noch keine revidierten ENETS-Leitlinien, aber die Guidelines von ENETS aus 2016 [16] und zu gastroenteropankreatischen NET von der European Society for Medical Oncology (ESMO) aus 2020 [17].

Bei lokalisierten und bei lokal fortgeschrittenen siNET empfehlen die ESMO-Leitlinien eine makroskopisch radikale Resektion mit mesenterialer Lymphadenektomie, um mitunter Komplikationen wie Obstruktion und Ischämie vorzubeugen [17]. Aus demselben Grund respektive zur Kontrolle eines Karzinoidsyndroms hat eine palliative/Debulking-Operation einen Stellenwert im metastasierten Stadium [17]. Für eine adjuvante Therapie gibt es keine Empfehlung bei siNET G1/G2 [17].

Hinsichtlich der medikamentösen Behandlung von siNET kann man zwei grundsätzliche Indikationen unterscheiden, die Symptomkontrolle und die Tumorkontrolle. Die Standardtherapie zur Kontrolle eines Karzinoidsyndroms sind die Somatostatin-Analoga Lanreotid und Octreotid in der monatlichen Depotinjektionsform, wobei bei Symptompersistenz/-verschlechterung eine SSA-Dosis-Intervallverkürzung (alle 2–3 Wochen), eine Add-on-Therapie mit dem Tryptophan-Hydroxylase-Inhibitor Telotristatethyl oder mit Interferon alpha sowie lebergerichtete Verfahren (RFA, TAE, TACE, SIRT) von den ESMO-Leitlinien empfohlen werden [17]. Zur Symptomkontrolle können auch PRRT und Everolimus beitragen, die erst bei Krankheitsprogression zum Einsatz kommen sollen [17]. Damit (SSA, PRRT, Everolimus) sind auch die zentralen Optionen für die Tumorkontrolle bei siNET genannt (Tab. 1).

Ein wichtiger Aspekt der Anti-Tumor-Therapie bei siNET ist, dass diese Therapien in der Regel die Krankheitsstabilisierung zum Ziel haben und nur in wenigen Fällen eine substanzielle Tumorverkleinerung bewirken können (partielles Ansprechen bei SSA und Everolimus in < 5 %, aber bei ca. 20 % mit PRRT) [21, 28, 25]. Auch wenn – wie oben angeführt – die SSA Lanreotid und Octreotid als gleich effektiv angesehen werden, gibt es hinsichtlich der Studienergebnisse doch nennenswerte Unterschiede: In der PROMID-Studie wurden 85 therapienaive Patient/-innen zu Octreotid oder Placebo randomisiert, und die mediane Zeit bis zur Tumorprogression (TTP) betrug 14,3 vs. 6 Monate (signifikant längere TTP; HR 0,34; 95 % KI 0,20–0,59) und das Gesamtüberleben 84,7 vs. 83,7 Monate (nicht signifikant) [21, 22]. Es ist beachtenswert, dass die TTP relativ kurz ist, wenn man bedenkt, dass die meisten eingeschlossenen Patient/-innen G1-Tumoren (fast nur Ki67 <2 %) und wenig Leber-Tumorlast hatten [21]. Im Gegensatz dazu wurden in CLARINET 204 Patient/-innen mit nichtfunktionellen enteropankreatischen NET G1/G2 mit einem Ki67 <10 %, mit vergleichsweise höherer Leber-Tumorlast und mit fast ausschließlich „stable disease“ eingeschlossen [5]. Das mediane PFS war in dieser Studie vergleichsweise lang (nicht erreicht vs. 18 Monaten, HR 0,47; 95 % KI 0,30–0,73) [5]. Entsprechend kann man laut Leitlinien SSA auch unabhängig vom Krankheitsstatus upfront verabreichen, und eine initiale Watch-and-wait-Strategie (bis zum dokumentierten Tumorprogress) ist bei indolenten Tumoren möglich, aber nicht notwendig [16, 17].

Die Phase-III-Studie NETTER‑1 hat PRRT plus Octreotid mit Hochdosis-Octreotid bei 229 Patient/-innen mit SSTR-positiven metastasierten Midgut-NET G1/G2 verglichen [25]. Das mediane PFS wurde nicht erreicht im PRRT-Arm vs. 8,4 Monate im Kontrollarm (HR 0,21; 95 % KI 0,13–0,33) [25]. Auch das mediane Gesamtüberleben (OS) war deutlich länger mit 48,0 Monaten vs. 36,3 Monaten, der Unterschied aber nicht signifikant (wie bei PROMID mitunter wegen der hohen Cross-over-Raten bzw. Folgetherapien) [26]. Die ESMO-Leitlinien empfehlen PRRT nach SSA, und wenn die Bedingungen für eine PRRT erfüllt sind [17].

Everolimus 10 mg/Tag wurde in der RADIANT-4-Studie bei metastasierten NET der Lunge und des gastrointestinalen Trakts mit Placebo verglichen (90 Lungen-NET und 175 gastrointestinale NET), wobei die Interventionsgruppe ein signifikant besseres PFS hatte (im Median 11,0 vs. 3,9 Monate; HR 0,48; 95 % KI 0,35–0,67) und der Vorteil auch in diesen beiden Subgruppen statistisch signifikant war [28]. Beachtenswert ist, dass etwa zwei Drittel im Everolimus-Arm Dosisreduktionen oder Therapieunterbrechungen hatten (vs. ein Drittel bei Placebo) und Nebenwirkungen von Grad 3–4 bei 1–10 % auftraten (Stomatitis, Diarrhoe, Infektionen, Pneumonitis und Hyperglykämie) [28]. Die ESMO-Leitlinien empfehlen Everolimus (unabhängig vom SSTR-Expressionsstatus) bei eindeutig progredienten siNET und nach einer PRRT – bei funktionellen siNET (Karzinoidsyndrom) ist der Nutzen aber weniger klar [17].

Weitere Optionen für Patient/-innen mit siNET sind in gewissen Situationen Interferon alpha (bei SSTR-negativen siNET) und Chemotherapie (CAPTEM und FOLFOX nur bei schnell wachsenden siNET G2-G3 mit hohem Ki67-Index, andernfalls ist Chemotherapie nicht indiziert) [16, 17].

## NET der Lunge

Lungen-NET („bronchopulmonary“, bpNET), also typische (TC) und atypische Karzinoide (AC), werden in der 2021 aktualisierten ESMO-Leitlinie behandelt [2]. Bei lokal begrenzten bpNET wird eine anatomische Resektion und Lymphknotendissektion empfohlen, wobei eine adjuvante Chemotherapie standardmäßig nicht vorgesehen ist [2]. Das Therapiespektrum bei den fortgeschrittenen oder metastasierten bpNET (Tab. 1) entspricht de facto dem der siNET, aber die Evidenzlage ist weitaus dürftiger. Zugelassen ist bei bpNET nur Everolimus infolge der RADIANT-4-Studie, die oben beschrieben ist (44 % der eingeschlossenen NET gingen von der Lunge aus) [28]. Für Lanreotid bei bpNET gibt es prospektive Evidenz von der randomisierten Phase-III-Studie SPINET, die aber aufgrund insuffizienter Rekrutierung frühzeitig gestoppt wurde [3]. Insgesamt wurden 77 Patient/-innen inkludiert, und das mediane PFS war sehr ähnlich zwischen der Lanreotid-Gruppe (16,6 Monate) und der Placebo-Gruppe (13,6 Monate) [3]. Die ESMO-Leitlinien empfehlen SSA als Erstlinientherapie bei langsam wachsenden bpNET, sofern ein SSTR-Rezeptorbesatz vorliegt, und Everolimus danach oder in der Erstlinie bei atypischen Karzinoiden [2].

Mitunter mangels anderer wirksamer Therapien, aber auch aufgrund ihres vergleichsweise günstigen Nebenwirkungsprofils wird die temozolomidbasierte Chemotherapie (meist CAPTEM als gänzliche oral Therapie) nach oder statt Everolimus (bei Unverträglichkeit oder bei aggressiveren Karzinoiden) empfohlen [2]. Retrospektive Analysen zeigen Ansprechraten von 20–30 % und ein medianes PFS um 10 Monate für CAPTEM bei bpNET [1]. Für die platinbasierte Chemotherapie gibt es auch positive retrospektive Daten, diese Therapie wird tendenziell als zweite Wahl empfohlen [2]. Weitere mögliche Optionen sind die PRRT (Phase-II-Daten) und Interferon alpha, wobei es dazu aktuell keine starke Evidenz gibt [2, 13].

Dieser Übersichtsartikel veranschaulicht das breite therapeutische Spektrum, das zur Behandlung von NET unterschiedlicher Lokalisation verfügbar ist. Aufgrund wesentlicher pathologischer und therapeutischer Unterschiede wurde die Gruppe der meist äußerst aggressiven neuroendokrinen Karzinome (NEC) hier nicht berücksichtigt. Oft steht das gesamte Therapieangebot nur in einem entsprechenden NET-Zentrum zur Verfügung und die Therapie sollte idealerweise interdisziplinär erfolgen. Schließlich ist es wichtig, gewisse NET-Patient/-innen auch im Rahmen klinischer Studien zu behandeln und einzuschließen, um diesen den Zugang zu potenziell wirksamen Therapien frühzeitig zu ermöglichen.

## Fazit für die Praxis

- Die endoskopische oder chirurgische Entfernung ist im lokalisierten Setting bei neuroendokrinen Tumoren (NET) potenziell kurativ.
- Für Somatostatin-Analoga (SSA) und Peptidrezeptor-Radionuklid-Therapie (PRRT) ist die Expression des Somatostatin-Rezeptors (SSTR) eine Voraussetzung.
- SSA sind in der Erstlinie bei gastroenteropankreatischen NET mit Ki67-Index < 10 % und bestimmten Lungen-NET zur Tumorkontrolle indiziert.
- Die besten Daten zur Wirksamkeit der PRRT gibt es bei Dünndarm-NET (von der Phase-III-Studie NETTER-1), aber es gibt auch Phase-II-Ergebnisse bei NET des Pankreas und der Lunge.
- Everolimus ist zur Therapie von gastrointestinalen, pankreatischen und pulmonalen NET mit progressiver metastasierter Erkrankung indiziert.
- Sunitinib und die Chemotherapie Streptozotocin/5-Fluoruracil sind weitere Optionen bei NET pankreatischen Ursprungs.
- Chemotherapie (temozolomid- oder platinbasiert) ist bei gewissen schnell wachsenden NET zu empfehlen.
